# Impact of secondary anterior-posterior defibrillator pad placement on chest compression interruptions: a three-arm randomised manikin-based simulation study among Dutch ambulance teams

**DOI:** 10.1016/j.resplu.2025.101064

**Published:** 2025-08-15

**Authors:** Freek Coumou, Dennie Wulterkens, Cornelis Slagt, Reinier Waalewijn, Lars Mommers

**Affiliations:** a Helicopter Emergency Medical Service Lifeliner 3, Nijmegen, the Netherlands; b Institute of Quality Medical Training Nieuwegein, the Netherlands; c Department of Anaesthesiology, Pain and Palliative Medicine, Radboud University Medical Centre, Nijmegen, the Netherlands; d Department of Cardiology, Gelre Hospitals, the Netherlands; e Department of Cardiology, St. Antonius Hospital, Nieuwegein, the Netherlands; f Department of Anaesthesiology and Pain Medicine, Maastricht University Medical Centre, Maastricht, the Netherlands; g Department of Simulation in Healthcare, Maastricht University Medical Centre, Maastricht, the Netherlands

**Keywords:** Paramedics, Ambulance, Out-of-hospital cardiac arrest, OHCA, Shockable arrest, Resuscitation, Defibrillation, Mechanical chest compression, Simulation

## Abstract

**Background:**

Out-of-hospital cardiac arrest management prioritises effective treatment, with high-quality chest compressions and timely defibrillation being essential. While current European Resuscitation Council guidelines recommend sternal-apical defibrillator pad placement, alternative positions such as anterior-posterior (AP) are gaining interest. The integration of secondary AP pad placement with mechanical cardiopulmonary resuscitation devices (mCPR) remains underexplored.

**Methods:**

This randomised, simulation-based study, assessed the impact of AP pad placement on chest compression interruptions, comparing manual versus mCPR strategies among 45 ambulance teams in the Netherlands. Teams were randomised into three groups: manual chest compressions with AP pad application, sequential mCPR and AP pad application, or simultaneous mCPR and AP pad application. The primary outcome was the duration of chest compression interruptions; secondary outcomes included user-feasibility and pad placement accuracy.

**Results:**

Applying AP pads during manual compressions required 38.3 ± 13.3 s, resulting in 12.1 ± 6.0 s of interruptions. Sequential mCPR and AP pad application took 97.7 ± 23.7 s, with 51.7 ± 14.0 s of interruptions. Simultaneous application required 70.5 ± 16.1 s and 31.8 ± 12.3 s of interruptions. Pad placement accuracy was low: 0 % for sternal, 11 % for apical, 13 % for anterior and 2 % for posterior pads. Participants’ confidence in pad placement did not correlate with accuracy.

**Conclusion:**

AP defibrillator pad placement during active mCPR presents significant challenges. Ambulance crews should assess the need for AP pads before initiating mCPR to minimise delays. The findings highlight substantial variability in pad placement, underscoring the need for standardised instructional guidelines and targeted training.

## Highlights

• mCPR substantially hindered anterior-posterior pad placement.
• Alternative pad placements should be considered before mCPR is applied.
• Experience with anterior-posterior pad placement is limited by ambulance crews.
• Only a minority of defibrillation pads were applied correctly by ambulance crew.
• Performance was not related to experience, self-evaluation or self-competence.

## Background

The management of out-of-hospital cardiac arrest (OHCA) with ventricular fibrillation is well-established within Advanced Life Support (ALS) guidelines, emphasizing the importance of early and effective treatment.^1^, ^2^, ^3^, ^4^ The duration of ventricular fibrillation is negatively correlated with successful outcomes, making high-quality chest compressions and early defibrillation crucial.^1^, ^5^ Contemporary ALS guidelines stress the importance of these interventions to maximize survival rates.^6^, ^7^

The current European Resuscitation Council (ERC) guidelines recommend initial defibrillation pads to be placed in the conventional sternal-apical (also commonly referred to as ‘anterior-lateral’) position, with the former pad placed ‘to the right of the sternum, below the right clavicle’ and the latter pad placed ‘in the left mid-axillary line, approximately level with the V6 ECG electrode’ and free of breast tissue.^7^ Alternative pad positions, such as the bi-axillary and anterior-posterior configurations are also described. Especially this latter anterior-posterior (AP) position is gaining interest, either as secondary position (‘vector change’) or as part of Dual Synchronized External Defibrillation (DSED) for persistent ventricular fibrillation,^8^, ^9^, ^10^, ^11^ The anterior pad is placed ‘anteriorly over the left precordium’ and the posterior pad is placed ‘posteriorly to the heart, just inferior to the left scapula’.^7^ Studies on the accuracy of these pad placements are scarce, especially when taking operational conditions such as weight and clothing into consideration.

Furthermore, limited personnel, rescuer fatigue and transportation can compromise high-quality chest compression in the prehospital setting.^12^, ^13^ To mitigate these issues, ambulance services in the Netherlands employ early mechanical CardioPulmonary Resuscitation (mCPR).^14^ The presence of mCPR might however hinder the application of a secondary set of anterior-posterior defibrillation pads.^15^ This study investigates the impact of manual versus mechanical CPR on *secondary* anterior-posterior pad placement in a simulation-based study methodology among ambulance ALS teams.

## Methods

### Trial design

This randomised, parallel-design study examined the impact of manual versus mechanical CPR on secondary anterior-posterior pad placement among ambulance teams in the Netherlands, using three different approaches. The allocation ratio between the groups was 1:1:1.

The study is described according to the extended CONSORT and STROBE guideline for simulation-based research.^16^ The checklist is available online (supplement A).

### Participants

All participants in this study were experienced emergency medical service (EMS) providers who underwent yearly ALS recertification and mCPR training as part of their ongoing professional recertification. Ambulance teams in the Netherlands are allowed to perform all standard ALS interventions, including manual defibrillation in the sternal-apical as well as anterior-posterior positions.

Inclusion criteria were set on active duty ambulance personnel with current certification in Advanced Life Support and proficiency in mCPR device operation. Each team consisted of two persons, one ambulance nurse (i.e. comparable to paramedic level) and one ambulance driver (i.e. comparable to EMT level). Teams were formed based on their usual working partnerships to ensure realistic crew dynamics.

### Interventions

The study utilized a simulated OHCA scenario with ventricular fibrillation. Ambulance crews were given a short introduction of the study but were blinded to the study hypothesis. Teams were given a short familiarization with the patient monitor and mCPR equipment.

Each session started with a single researcher (FC) performing chest compressions only on the manikin. This ‘BLS-researcher’ was also ALS certified. No ventilations were performed during this study. Ambulance crews were instructed to respond as they would to an actual OHCA. They were explicitly instructed to handle the manikin’s clothing as they would do in a real-life situation.

**Fig. 1:**
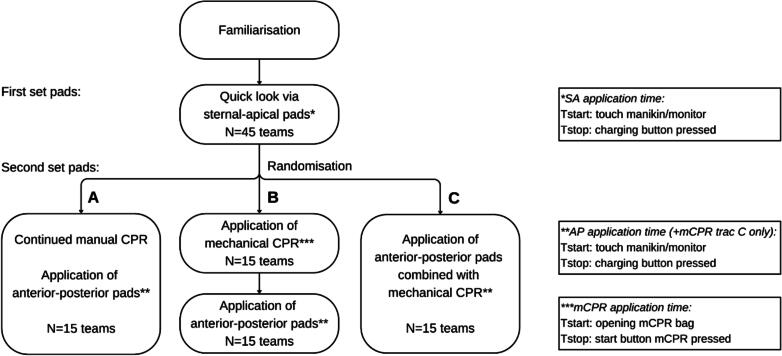
Flowchart of study: Team numbers, randomisation, and treatment pathways.

All teams performed an initial 'quick look' cardiac rhythm assessment through placement of sternal-apical defibrillation pads. Hereafter, ambulance crews were randomized to one of three AP pad application sequences (Fig. 1):

### Group A, manual chest compression, application of AP-pads

The BLS-researcher continued chest compressions while the ambulance crew applied the second set of AP defibrillation pads.

### Group B, sequential mCPR and AP-pads application

The BLS-researcher continued chest compressions until the ambulance crew applied their mCPR device. Once mechanical chest compressions were established, a third assignment was given to apply AP pads, necessitating the interruption of mCPR.

### Group C, simultaneous application of mCPR and AP-pads

The BLS-researcher continued chest compressions until the ambulance crew applied their mCPR device concurrently with AP defibrillation pads.

No immediate debriefing was conducted. Feedback was withheld from participants to avoid influencing subsequent teams.

### Outcomes

Primary outcome was the duration of chest compression interruptions with anterior-posterior defibrillator pad application measured in seconds. The following predetermined time intervals were measured: ‘*defibrillator pad application time*’ starting when ambulance crew touched either the monitor or the manikin after the given assignment and stopped when the defibrillator charging button was pressed, measured in seconds. ‘*mCPR application time*’ started when EMS opened the mCPR bag and stopped when mechanical chest compressions were started, measured in seconds. Secondary outcomes were perceived user-feasibility of the three different approaches and accurateness of the placed defibrillation pads.

### Establishment of optimal pad placement

Two expert resuscitation specialists independently determined the optimal placement for all four defibrillation pads (sternal, apical, anterior and posterior) in accordance with the current resuscitation guideline.^7^ Each expert performed two separate placements of all four pads at least one week apart. Measurements were taken for centre point coordinates and angles of pad orientation and averaged into an expert consensus reference position. A grid system was established on the manikin using permanent markings not recognizable by the study participants. Further details can be found in our earlier publication.^17^

Centre points were marked as correct when placed within a 50 mm diameter around the reference centre points. Angles were marked as correct when deviations remained within 45 degrees from the reference angle. Pads were marked as ‘correctly placed’ when both criteria were met.

### Sample size

A total of 45 ambulance ALS teams were recruited for this study, with 15 teams allocated to each of the three intervention groups. The sample size was calculated using R with the TrialSize package, based on the formula from Chowet al. (2017)^18^ for sample size calculations in clinical research. The calculation assumed a medium effect size (Cohen's f = 0.25), α = 0.05, and power (1-β) of 0.80 for a one-way ANOVA with three groups, under the assumption of no dropouts.

### Randomization

Randomisation was achieved using a sealed envelope system, where each envelope contained the allocation for one of the three groups (A, B, or C). The envelopes were randomly drawn for each team, ensuring that the allocation was concealed until the point of assignment. Fifteen teams were assigned to each group, without any cross overs.

### Data collection and blinding

Primary outcome was captured using high-definition cameras positioned to capture the entire resuscitation area and a digital clock. This approach enabled precise measurement of time intervals and interruptions to chest compressions. The video recordings were analysed by two reviewers (DW, FC). To ensure data integrity, a random 20 % sample of videos was re-analysed for the primary outcome by a third reviewer (CS) blinded to the initial results. Any discrepancies greater than 5 % were resolved through consensus discussion.

Secondary outcome on pad positioning was assessed by a single researcher (DW) measuring the distances from each pad’s centre point to the reference centre point as well as the angle of pad orientation. A second researcher (FC) independently verified all measurements using standardised photographs.

Self-reported feasibility was collected via a QR code-based survey created using the Qualtrics online research platform (Qualtrics™, Provo, UT, USA). The survey comprised three demographic questions, four multiple-choice experience questions, and eight Likert-scales questions (supplement B).

### Equipment

The REALITi360 Plus monitor (iSimulate, now 3B Scientific, Germany) was utilized as the patient monitor and defibrillator. This device allows for customization of the user interface to mimic the appearance and functionality of various monitors commonly used by ambulance services, including Philips, Zoll, Lifepak, Corpuls, and Medtronic. Two sets of self-adhesive defibrillation pads were available, sized 88x132mm, with 1.5 m cable length.

A CrashTest-Service® rescue manikin from the fire and extrication department was used to resemble more realistic human body dimensions and weight. The length of the manikin was 172 cm with an abdominal and chest circumference of 99 and 102 cm respectively. The manikin weighted 90 kg. The manikin was dressed in a pair of trousers and two layers of upper clothing.

The mCPR device was provided by the ambulance service and made available near the manikin within the original storage bag.

### Statistical analysis

Quantitative data were analysed using IBM Statistic Package for the Social Sciences (IBM Corp.© SPSS Statistics for Windows, Version 28.0.0.0). Continuous variables (time) were reported as mean ± SD. Dichotomous variables (correctness of pad position) were reported as counts (N) and percentages (%). Differences between groups were analysed using an independent sample *t*-test (two groups) or one-way ANOVA (three or more groups). Associations between variables were tested using Spearman’s rho test.

### Recruitment

Participants were recruited at various ambulance stations to ensure a homogeneous group, with data collection occurring between May 2024 and January 2025. Teams were selected based on their availability on the schedule, ensuring that no team was included more than once in the study.

## Ethics

All participants were informed about the study prior to data collection and had the opportunity to ask questions before they provided informed consent. All teams participated voluntarily and could withdraw their consent at any time during the study. All data were collected anonymously. The METC Oost-Nederland declares that the research does not fall under the WMO (2024-17264). Therefore, no positive assessment is required from the METC Oost-Nederland or another recognized medical-ethical review committee for its implementation. The study complies with the Declaration of Helsinki.

## Results

**Fig. 2:**
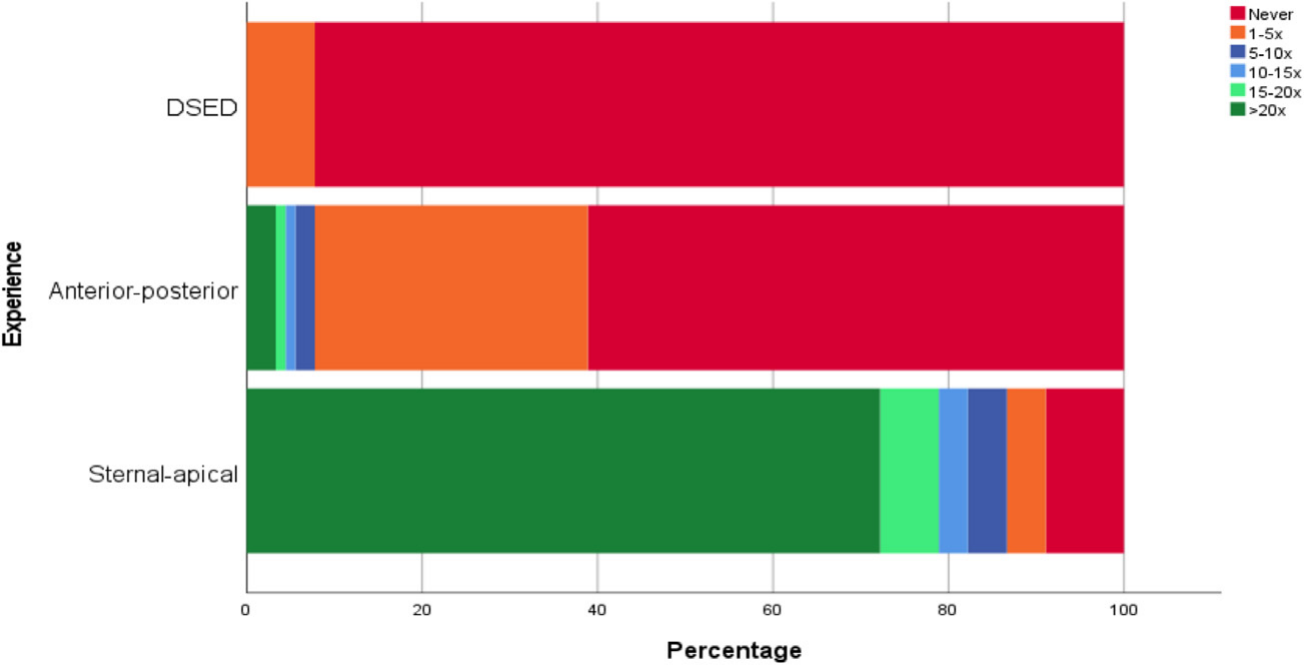
Experience with standard and alternative defibrillation positions among participants (*N* = 90).

A total of 90 individual participants, 45 ambulance teams, were recruited from four ambulance regions in the Netherlands. Demographic characteristics of the participation ambulance crew and their levels of experience with alternative defibrillation strategies are presented in Table 1 and Fig. 2, respectively.

**Table 1:** Participants’ baseline demographics (*N* = 90).

**Gender**	**N (%)**
Male	62 (68.9)
Female	28 (31.1)
**Age**	
Mean ± SD yrs	42.6 ± 9.9

**Background**	
Ambulance (registered) nurse	43 (47.8)
Ambulance driver	42 (46.7)
Bachelor Emergency Medicine	4 (4.4)
Physician assistant	1 (1.1)

**Clinical experience**	
0–5 years	20 (22.2)
6–10 years	19 (21.1)
11–15 years	17 (18.9)
16–20 years	14 (15.6)
>20 years	20 (22.2)

Among the ambulance teams, 36 (80 %) utilized a Corpuls3 patient monitor, while the remaining 9 teams (20 %) employed a Lifepak15 monitor. The majority of teams, 28 (62.2 %), used the LUCAS3 mechanical CPR (mCPR) device for chest compressions, whereas 17 teams (37.8 %) used the CorpulsCPR device.

The mean ± SD procedure time for the initial quick look in the sternal-apical position was 37.5 ± 10.7 s, with a chest compression interruption of 5.4 ± 6.0 s. Applying anterior-posterior pads while maintaining manual chest compressions (group A) required a mean time of 38.3 ± 13.3 s, resulting in 12.1 ± 6.0 s of chest compression interruptions. The time needed to apply mCPR was 57.7 ± 14.0 s, with 21.5 ± 9.0 s of chest compression interruptions. For participants who had previously applied mCPR (Group B), the additional time required to apply anterior-posterior pads was 40.0 ± 14.0 s, with 30.1 ± 15.0 s of chest compression interruptions. The combined approach of applying mCPR and anterior-posterior pads simultaneously (group C) required a mean procedure time of 70.5 ± 16.1 s, with 31.8 ± 12.3 s of chest compression interruptions; these results are detailed in Table 2. Total chest compression interruptions were significantly different between the three groups, Welch’s F(2, 24.1) = 21.0, *p* < 0.001. Groups B and C had significant longer interruptions for AP pad application as compared to group A with an increase of 18.1sec (95 %CI: 7.4–28.7), *p* = 0.001 and 19.7sec (95 %CI 10.8–28.7), *p* = <0.001 respectively. When comparing total interruptions between both mCPR groups, group C had less interruptions with a difference of 19.9 s (95 % CI 7.3–32.5 s), t(28) = 3.23, p = 0.003.

**Table 2:** Defibrillation pad application times and BLS interruptions per intervention group.

**Sternal-apical procedure**	**Quick look assessment**			
Number of teams (N)	45 teams			
Procedure time (sec), mean±SD	37.5 ± 10.7			
Number of chest compression interruptions (N), mean±SD	1.4 ± 1.0			
Duration chest compression interruption (sec), mean±SD	5.4 ± 6.0			

**Antero-posterior procedure**	**Group A**	**Group B**	**Group C**	**Significance (p)**

Number of teams (N)	15 teams	15 teams	15 teams	

Total procedure time (sec), mean±SD	38.3 ± 13.3	97.7 ± 23.7	70.5 ± 16.1	*P* < 0.001
mCPR application only	N/A	57.7 (14.0)	Combined	N/A

Number of chest compression interruptions (N), mean ± SD	1.7 ± 0.6	2.8 ± 0.8	2.4 ± 0.8	*P* < 0.001
mCPR application only	N/A	1.4 ± 0.5	Combined	N/A

Duration chest compression interruptions (sec), mean±SD	12.1 ± 6.0	51.7 ± 20.4	31.8 ± 12.3	*P* < 0.001
mCPR application only	N/A	21.5 (9.0)	Combined	N/A

Perceived user effects*				
Number of participants (N)	30	30	30	
Feasibility (mean±SD)	8.3 ± 1.3	7.4 ± 2.0	7.7 ± 1.6	*P* = 0.11
Speed (mean±SD)	8.2 ± 1.1	6.9 ± 2.0	7.0 ± 1.9	*P* = 0.003
Patient benefit (mean±SD)	6.5 ± 1.7	6.3 ± 2.2	6.5 ± 1.9	*P* = 0.91
Self-competence (mean±SD)	8.0 ± 2.0	7.7 ± 2.2	7.9 ± 1.9	*P* = 0.80

* 11-item Likert scale (0–10).

**Fig. 3:**
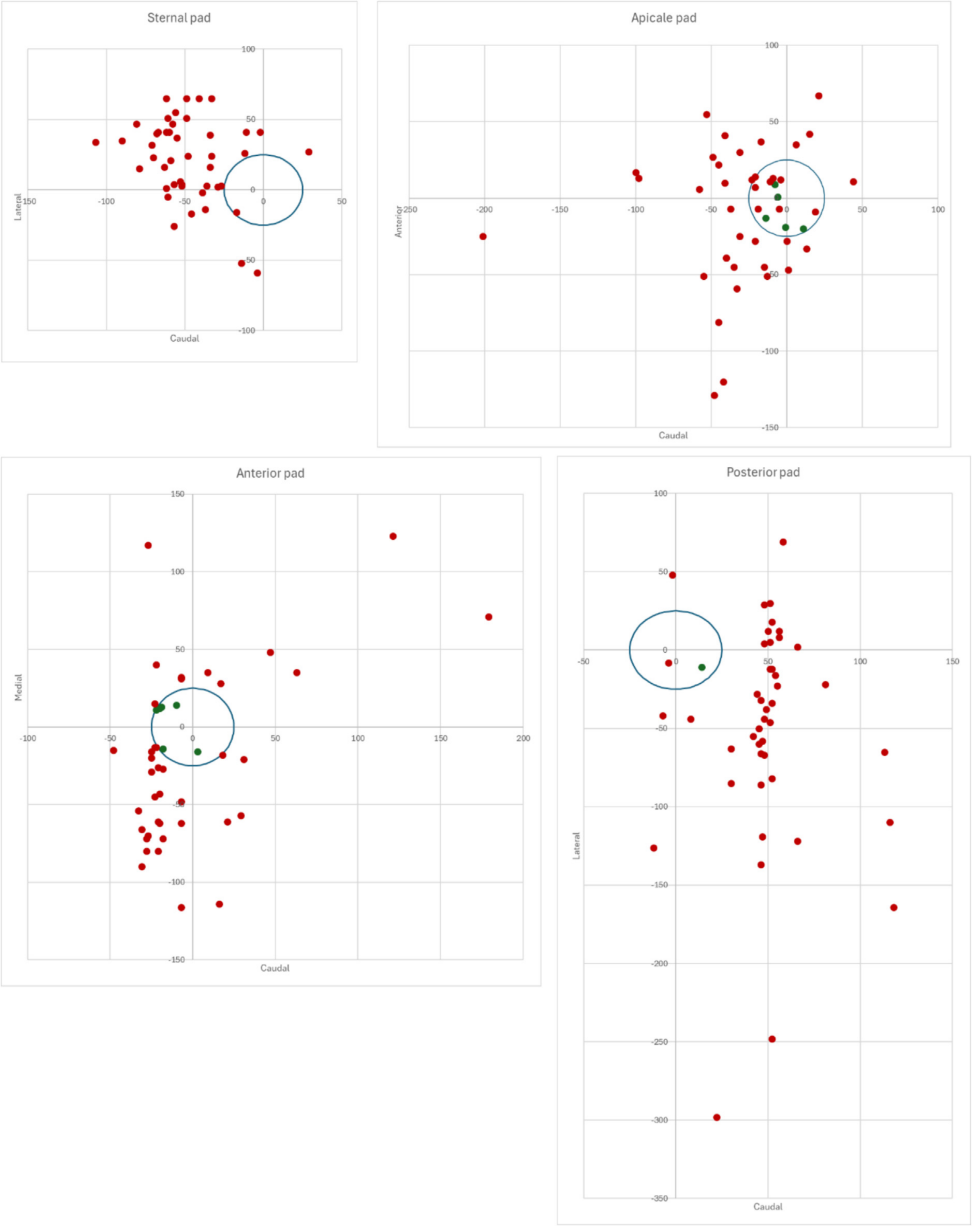
Distribution of pad placements for the sternal-apical and antero-posterior positions (*N* = 45 per position). **Legend**: The figures depict the placement of pads relative to the reference, with distances measured in millimetres. Green dots indicate correct placement, while red dots signify incorrect placement (centre point outside the blue reference circle) or an angle exceeding the reference measurement. (For interpretation of the references to colour in this figure legend, the reader is referred to the web version of this article.)

The accuracy of defibrillator pad placement by position is summarized in Table 3 and Fig. 3. Correct placement was observed in only a small proportion of pads: none (0 %) in the sternal position, 5 (11 %) in the apical position, 6 (13 %) in the anterior position, and 1 (2 %) in the posterior position. The majority of sternal pads were placed too laterally (*N* = 38, 84 %) and/or too cranially (*N* = 22, 49 %). For apical pads, 20 (44 %) were positioned too anteriorly and 14 (31 %) too caudally; additionally, 28 (62 %) were not aligned longitudinally. Among anterior pads, 21 (47 %) were placed too caudally. Similarly, posterior pads were frequently misplaced, with 38 (84 %) placed too medially and 27 (60 %) too caudally.

**Table 3:** Accurateness of defibrillator pad placement in four different positions.

**Position**	**Sternal**	**Apical**	**Anterior**	**Posterior**
Number of pads applied, N (%)	45 (100 %)	45 (100 %)	45 (100 %)	45 (100 %)
Correct placement, N (%)	0 (0 %)	5 (11 %)	6 (13 %)	1 (2 %)

Angle fault, N (%)	19 (42 %)	28 (62 %)	6 (13 %)	9 (20 %)
Centre point fault, N (%)	44 (98 %)	33 (73 %)	39 (87 %)	43 (96 %)
Too cranial	22 (49 %)	8 (18 %)	10 (22 %)	4 (9 %)
Too caudal	3 (7 %)	14 (31 %)	21 (47 %)	27 (60 %)
Too lateral / posterior	38 (84 %)	1 (2 %)	6 (13 %)	0 (0 %)
Too medial / anterior	1 (2 %)	20 (44 %)	8 (18 %)	38 (84 %)
Distance to reference mean±SD, mm	61.1 ± 20.4	50.3 ± 37.8	58.1 ± 38.4	84.7 ± 55.8
Distance to reference min, mm	23	6	16	9
Distance to reference max, mm	112	203	193	299

Participants rated their confidence in the correct placement of defibrillator pads on a scale from 0 (extremely uncertain) to 10 (extremely certain). The mean ± SD confidence scores were 8.3 ± 1.3 for sternal-apical pads and 7.3 ± 1.8 for anterior-posterior pads. However, Spearman’s rho analysis revealed no significant correlation between self-perceived confidence and actual performance for either sternal-apical pads (*ρ* = 0.178, *p* = 0.10) or anterior-posterior pads (ρ = −0.048, *p* = 0.66).

Participants were also surveyed about their ambulance service's policies regarding the use of anterior-posterior pad placement and DSED in cases of persistent ventricular fibrillation. A significant proportion of respondents were unaware of these policies: 61.8 % (*N* = 55) for anterior-posterior placement and 68.5 % (*N* = 61) for DSED. Among those who responded definitively, 23.6 % (*N* = 21) reported that their service permitted anterior-posterior placement, while 14.6 % (*N* = 13) stated it was not permitted. For DSED, 7.9 % (*N* = 7) reported it was allowed, and 23.6 % (*N* = 21) denied permission.

## Discussion

This study found considerable variability in chest compression interruptions associated with the application of secondary anterior-posterior defibrillation pads. Manual chest compressions resulted in the fewest interruptions, followed by the simultaneous application of mCPR and AP pads. Sequential application of mCPR and AP pads was associated with the longest procedure time and the most prolonged interruption in chest compressions. The accuracy of defibrillator pad placement was low across all positions.

The time required to apply mCPR in our study is comparable to that reported in other studies,^19^, ^20^ although continuous quality improvement initiatives have been shown to reduce application times.^20^, ^21^, ^22^ The procedure times observed in our study are relatively long due to our definitions: we deliberately included the ‘preparation’ phase for both mCPR and secondary defibrillation pads, as this occupies an ambulance team for a certain period. Procedure time is most relevant for *primary interventions* (e.g. initial rhythm check). For *secondary interventions,* as examined in this study, preparations can be made during a 2-min CPR cycle. We therefore focused primarily on interruptions of chest compressions. The interruptions of chest compression observed during mCPR application in our study are comparable to those reported in real-life mCPR scenarios.^23^, ^24^ This suggests that the mannikin’s body weight and clothing contributed to a training experience comparable with real life situations.

The study commenced intentionally with sternal-apical pad placement, as it remains the standard practice for quick-look rhythm analysis in the Netherlands.^7^ The sternal-apical approach in this study underscores this with fewer interruptions compared to anterior-posterior pad placement. However, additional research is warranted to determine the clinical significance of these interruption differences. For example, the study by Luptonet al. (2024)^11^ compared initial sternal-apical versus anterior-posterior pad placement in a matched observational cohort and observed higher rates of return of spontaneous circulation in the latter group.

Most critically, our study highlights the adverse impact of mCPR devices on AP defibrillation pad placement. Sequential application (Group B) required vast time investment and resulted in the longest chest compressions interruption. The additional time needed to pause mechanical chest compressions and apply AP pads in Group B was nearly equivalent to the total application time for simultaneous mCPR and AP pad placement (Group C). This demonstrates that AP defibrillation pad placement becomes significantly more challenging once mCPR is initiated. Emergency health care providers should therefore carefully consider the potential need for AP pads before initiating mCPR. Where feasible, AP pads should be applied prior to or concurrently with mCPR activation.

The low accuracy of defibrillator pad placement observed in this study aligns with prior research highlighting the challenges of achieving correct positioning.^17^, ^25^, ^26^, ^27^, ^28^, ^29^ The use of a manikin with a realistic weight, in combination with time constraints and the presence of actual clothing, likely exacerbated these difficulties and resulted in even lower accuracy rates. However, these low accuracy percentages should not be interpreted as indicative of poor clinical performance, nor should they be regarded as grounds to postpone or avoid defibrillation practice! Clinical research correlating pad placement with patient outcomes remains limited and further studies are needed to clarify the relationship between pad position, transcardiac current vector and clinical efficacy.^30^, ^31^

Instead, these results should be interpreted as evidence for the inherent complexity of defibrillator pad placement. Critical questions remain, such as whether energy selection can compensate for placement deviations and if survival rates might improve with more precise positioning. Meanwhile, the observed deviations of 50–85 mm (with maxima approaching 300 mm) represent a clinically significant challenge.

Additionally, despite established guidelines emphasising the importance of longitudinal orientation for apical pad placement,^7^, ^32^ nearly two-thirds of apical pads were positioned incorrectly (i.e. outside a 45 degree angle), highlighting the challenge of translating guidelines into consistent clinical practice. Meanwhile, whether energy selection and/or waveform adjustments can compensate for vector deviations – and up to what degree of deviation such compensation remain effective – has not yet been fully elucidated. A recent animal study by Esibovet al. (2016)^33^ is particularly noteworthy, demonstrating a substantial negative impact of minor (<30 mm) deviations in electrode pad placement on defibrillation success. Importantly, the authors also showed that both waveform design and energy selection have the potential to overcome these minor misplacements.^33^

Beyond technical compensation, the variability in performance observed in this study underscores the need for improved user training. However, given that similar variability exists among instructors themselves, there is a clear need for targeted ‘train-the-trainer’ programmes alongside uniform clear and standardised instructional materials for healthcare professionals as well as lay persons.^17^, ^34^ Finally, a notable proportion of respondents reported uncertainty regarding their organisation’s policy on alternative defibrillation positions, suggesting an opportunity to enhance targeted education and protocol dissemination.^17^

Variability in clothing removal techniques was observed among teams, with methods including tearing, cutting, ripping, or sliding garments aside. Most teams opted to cut or tear clothing along the midline- a strategy that, while potentially faster (e.g. for buttoned shirts), often left excess fabric on the sides. This residual material could impede the placement of lateral and posterior pads, as well as the application of mCPR devices such as the LUCAS3. Further variability was observed in the placement of the posterior pad, with teams either log-rolling the patient or having them sit up. Additionally, variability in procedural roles was noted: some teams delegated pad application to the ambulance driver—positioned at the patient’s side—while others assigned it to the ambulance nurse—positioned at the patient’s head. Previous research among laypersons has found no significant difference in preferred sides for applying automated external defibrillator (AED) pads, with apical pad deviations averaging 59 mm (left side) and 69 mm (right side).^28^ As this study was not designed to explore these variables and given the overall low accuracy of pad placement, further analysis of such factors was not feasible.

## Limitations

This study's assessment of defibrillator pad placement accuracy was based on expert consensus informed by contemporary resuscitation guidelines. While expert consensus complements guideline recommendations, we acknowledge that criteria for ‘correct’ placement remain debatable. To enhance reliability, a dual-expert repeated measurement approach was adopted, with placement criteria derived from prior studies.^17^, ^25^, ^27^, ^32^ Centre-point tolerances previously used are relatively strict (50 mm radius), while angular allowances are rather broad − up to 45 degrees. Research on the clinical implications of defibrillation pad deviations, however, remains limited, restricting the extrapolation of our findings to patient outcomes.

We deliberately excluded ventilations by ambulance crews to isolate the impact of pad placement on procedural timings and to avoid confounding interruptions. This approach reflects real-world practice, where interventions such as alternative pad positioning are typically prepared during 2-min CPR cycles and executed during ventilatory pauses to maximise chest compression fraction.

The embedded researcher’s provision of high-quality chest compressions may have reduced participants’ cognitive load, potentially improving their focus on pad placement. While often additional crew members are available, it could introduce bias by simplifying task execution compared to real-world, resource-constrained settings. The same consideration applies to other factors such as psychological pressure, physical fatigue, concurrent task demands and anatomical variations^35^ which were absent in this simulation.

## Conclusion

This simulation study highlights the significant challenges of anterior-posterior defibrillator pad placement during active mCPR. Ambulance crews should prioritise assessing the need for anterior-posterior pads before initiating mCPR to minimise delays and interruptions to chest compressions. The findings also reveal substantial variability in pad application accuracy, underscoring the need for standardised instructional guidelines and targeted training programmes. Future research should explore strategies to optimise defibrillator pad placement in real-world settings, where factors such as anatomical variations and operational stressors may further influence outcomes.

## CRediT authorship contribution statement

**Freek Coumou:** Investigation. **Dennie Wulterkens:** Investigation. **Cornelis Slagt:** Writing – review & editing. **Reinier Waalewijn:** Writing – review & editing. **Lars Mommers:** Writing – original draft, Formal analysis, Conceptualization.

## Funding

This research did not receive any specific grant from funding agencies in the public, commercial, or not-for-profit sectors.

## Declaration of competing interest

The authors declare that they have no known competing financial interests or personal relationships that could have appeared to influence the work reported in this paper.
