# Supplementary material for: Impact of secondary anterior-posterior defibrillator pad placement on chest compression interruptions: a three-arm randomised manikin-based simulation study among Dutch ambulance teams
Source: Resusc Plus. 2025 Aug 15;26:101064. doi: 10.1016/j.resplu.2025.101064 (PMC12410578; doi:10.1016/j.resplu.2025.101064)
Supplement: Supplementary Data 2 [file mmc2.docx]

**Ambulance team survey**

- Informed consent:
- I have been able to read the information (letter).
- Any questions I had have been sufficiently answered.
- I had enough time to decide about participation.
- I know that participation is voluntary and that I can stop at any time.
- I know that if I stop, my data up to that point may be used.
- I give permission for the collection, storage, and use of anonymous research data.
- I agree to participate in this study:

Agree to participate (1)

**What is your age?**

**Please select your gender:**

Man

Woman

Other (please specify)

**Please select your background:**

Ambulance nurse

Ambulance driver

BMH (Bachelor of Medical Assistance)

Other (please specify)

**Please select the years of clinical Advanced Life Support experience you have:**

0–5 years

6–10 years

11–15 years

16–20 years

>20 years

**Please select how often you personally have performed standard (antero-lateral) defibrillation?**

Never

1–5 times

5–10 times

10–15 times

15–20 times

>20 times

**Please select how often you personally have performed front-back (antero-posterior) defibrillation?**

Never

1–5 times

5–10 times

10–15 times

15–20 times

>20 times

**Please select how often you personally have performed dual synchronized external defibrillation (DSED)?**

Never

1–5 times

5–10 times

10–15 times

15–20 times

>20 times

**Is it permitted within your Regional Ambulance Service to use (single) front-back defibrillation for persistent VF?**

Yes

No

I don't know

**Is it permitted within your Regional Ambulance Service to use Dual Synchronized External Defibrillation (DSED) for persistent VF?**

Yes

No

I don't know

**How confident are you that the standard (antero-lateral) pads are placed correctly on the manikin?**

0 = extremely uncertain, 5 = neutral, 10 = extremely certain

**How confident are you that the front-back (antero-posterior) pads are placed correctly on the manikin?**

0 = extremely uncertain, 5 = neutral, 10 = extremely certain

**To what extent do you (dis)agree with the following statements?**

**The secondary defibrillation sequence just performed is technically feasible:**
0 (completely disagree) – 5 (neutral) – 10 (completely agree)

**The secondary defibrillation sequence just performed can be carried out quickly:**0 (completely disagree) – 5 (neutral) – 10 (completely agree)

**The secondary defibrillation sequence just performed has added value for the patient:**0 (completely disagree) – 5 (neutral) – 10 (completely agree)

**I feel competent that I can apply this defibrillation sequence in practice:**
0 (completely disagree) – 5 (neutral) – 10 (completely agree)

**Space for optional (final) comments:**

_________________________________________________

Thank you very much for completing this survey and for your cooperation in this research.

Disclaimer: the survey was translated from Dutch to English for publication without formal backtranslation and verification.
